# MRI-Only Based Radiotherapy Treatment Planning for the Rat Brain on a Small Animal Radiation Research Platform (SARRP)

**DOI:** 10.1371/journal.pone.0143821

**Published:** 2015-12-03

**Authors:** Shandra Gutierrez, Benedicte Descamps, Christian Vanhove

**Affiliations:** Medical Image and Signal Processing Group, Ghent University-iMinds Medical IT department, Ghent, Belgium; Shenzhen institutes of advanced technology, CHINA

## Abstract

Computed tomography (CT) is the standard imaging modality in radiation therapy treatment planning (RTP). However, magnetic resonance (MR) imaging provides superior soft tissue contrast, increasing the precision of target volume selection. We present MR-only based RTP for a rat brain on a small animal radiation research platform (SARRP) using probabilistic voxel classification with multiple MR sequences. Six rat heads were imaged, each with one CT and five MR sequences. The MR sequences were: T1-weighted, T2-weighted, zero-echo time (ZTE), and two ultra-short echo time sequences with 20 μs (UTE1) and 2 ms (UTE2) echo times. CT data were manually segmented into air, soft tissue, and bone to obtain the RTP reference. Bias field corrected MR images were automatically segmented into the same tissue classes using a fuzzy c-means segmentation algorithm with multiple images as input. Similarities between segmented CT and automatic segmented MR (ASMR) images were evaluated using Dice coefficient. Three ASMR images with high similarity index were used for further RTP. Three beam arrangements were investigated. Dose distributions were compared by analysing dose volume histograms. The highest Dice coefficients were obtained for the ZTE-UTE2 combination and for the T1-UTE1-T2 combination when ZTE was unavailable. Both combinations, along with UTE1-UTE2, often used to generate ASMR images, were used for further RTP. Using 1 beam, MR based RTP underestimated the dose to be delivered to the target (range: 1.4%-7.6%). When more complex beam configurations were used, the calculated dose using the ZTE-UTE2 combination was the most accurate, with 0.7% deviation from CT, compared to 0.8% for T1-UTE1-T2 and 1.7% for UTE1-UTE2. The presented MR-only based workflow for RTP on a SARRP enables both accurate organ delineation and dose calculations using multiple MR sequences. This method can be useful in longitudinal studies where CT’s cumulative radiation dose might contribute to the total dose.

## Introduction

Small animal models are widely used for the development of new cancer treatment strategies. The recently developed 3D conformal small animal micro-irradiators have significantly reduced the technological gap between laboratory radiation research and human treatment methods [1,2]. These devices are using kV X-ray sources that combine irradiation with high-resolution cone-beam (CB) computed tomography (CT) [3]. However, CB-CT is hampered by low soft-tissue contrast [4] making accurate target selection very difficult, especially in the rodent brain. In contrast, magnetic resonance (MR) images offer excellent soft-tissue contrast but they do not provide the required electron density information needed for dose calculation [5–8].

The aim of this study is to investigate the feasibility of an MR-only based workflow for rat brain radiotherapy treatment planning (RTP) on a small animal radiation research platform (SARRP) that enables both accurate target delineation and accurate dose calculations. Therefore, the MR volume needs to be segmented into a limited number of tissue classes. Electron density values have to be assigned to these classes to override their default MR signal intensity values. However, tissue segmentation in MR imaging is far from trivial. Conventional MR sequences provide no signal in lung and bone, due to the low proton densities and very short transverse relaxation times of these tissue types. As a result, there is no contrast between air, lung and bone. To solve this problem, novel MR sequences have been developed that acquire the MR signal directly after radio-frequency excitation, such as ultra-short echo time (UTE) and/or zero echo time (ZTE) sequences. The extra information provided by such sequences can be used to facilitate segmentation of the MR images [9–12]. To generate segmented MR images, we employ probabilistic classification of voxels using multi-sequence MR images [13]. To our knowledge, this is the first study that investigates the use of an MR-only based workflow in pre-clinical RTP and the first study that uses ZTE sequence for image segmentation.

## Materials and Method

### Animals

**Fig 1:**
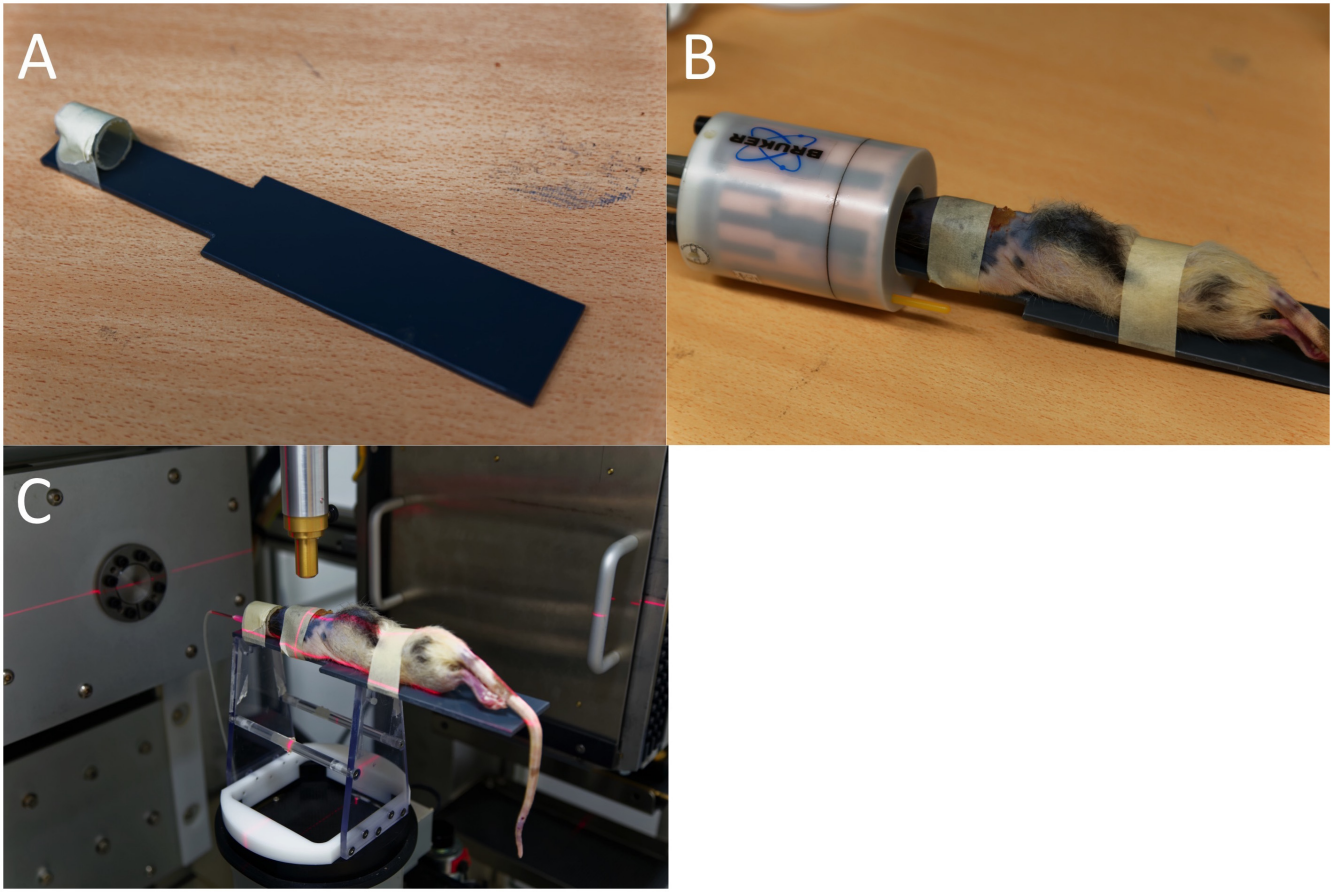
Homemade multi-modality bed. a) our homemade bed, b) the rat position on the bed in front of the MR volume coil, c) the rat position on the bed in the SARRP.

Six adult female Fisher rats (weight: 174±7g) were purchased from Charles River (Neder-over-Heembeek, Belgium). All animals were treated according to the European Ethics Committee (2010/63/EC). All experimental procedures were approved by the Animal Experimental Ethical Committee of Ghent University Hospital (ECD 12/28-A1). The animals were kept under environmentally controlled conditions (12h normal light/dark cycles, 20–23°C, 50% relative humidity) with food and water ad libitum. During the imaging experiment, animals were anesthetized with 2% isoflurane mixed with medical oxygen (0.3L/min). To facilitate animal transport from one imaging device to another and to simplify image co-registration, rats were positioned on an in-house made multi-modality bed to maintain a fixed rat head position (Fig 1). After completion of the experiment the animals were euthanized with an overdose of pentobarbital (150mg/kg, i.p.).

### MR Image Acquisition

MR images of the rat head were acquired on a 7 Tesla system (Bruker PharmaScan 70/16, Ettlingen, Germany) using a 40 mm Bruker quadrature volume transmit/receive radiofrequency coil. Five different MR sequences were acquired for each animal (Table 1). Anatomical images of the rat head were collected using a T1-weighted Modified Driven Equilibrium Fourier Transform (MDEFT) sequence and a T2-weighted Rapid Acquisition with Relaxation Enhancement (RARE) sequence. In addition to these structural images, three other MR sequences were acquired, including an ultra-short echo time sequence with 20μs echo time (UTE1), an ultra-short echo time sequence with 2ms echo time (UTE2) and a zero echo time sequence (ZTE). UTE and ZTE offer the opportunity to acquire images from proton-poor structures with very short transverse relaxation times, such as bone, by using a rapid readout of the fast decaying signal. All MR images were acquired in coronal orientation.

**Table 1:** Overview of the acquisition parameters used for the five MR sequences.

	MDEFT-T1	RARE-T2	UTE1	UTE2	ZTE
**TR (ms)**	1700	16000	8	8	2
**TE (ms)**	3.5	37	0.02	2	0
**TI (ms)**	1100	-	-	-	-
**FA (°)**	20	164.4	7.5	7.5	1.34
**NA**	1	3	3	3	12
**TA (min)**	29	12	20	20	20
**Matrix**	128^3^	128^3^	128^3^	128^3^	128^3^
**Voxel size(μm)**	275x500x275	275x500x275	275x500x275	275x500x275	275x500x275

TR: Repetition time, TE: Echo time, TI: Inversion time, FA: Flip angle, NA: Number of averages, TA: Total acquisition time.

### Bias Field Correction

A bias field is a low frequency undesirable smooth signal that corrupts MR images due to inhomogeneities in the scanner’s field strength. These low level variations degrade the performance of segmentation algorithms that use individual voxel gray level values. Consequently, a preprocessing step is required to correct for bias field effect before doing segmentation. Bias field correction was done using a 3D extended version of Coherent Local Intensity Clustering (CLIC) algorithm presented by Li et al [14]. In this retrospective algorithm bias field correction and tissue classification are interleaved to benefit from each other, thereby allowing both to be refined iteratively until convergence to an optimal solution. In this study only the estimated bias field was used to correct for the MR inhomogeneities.

### CT Image acquisition

**Fig 2:**
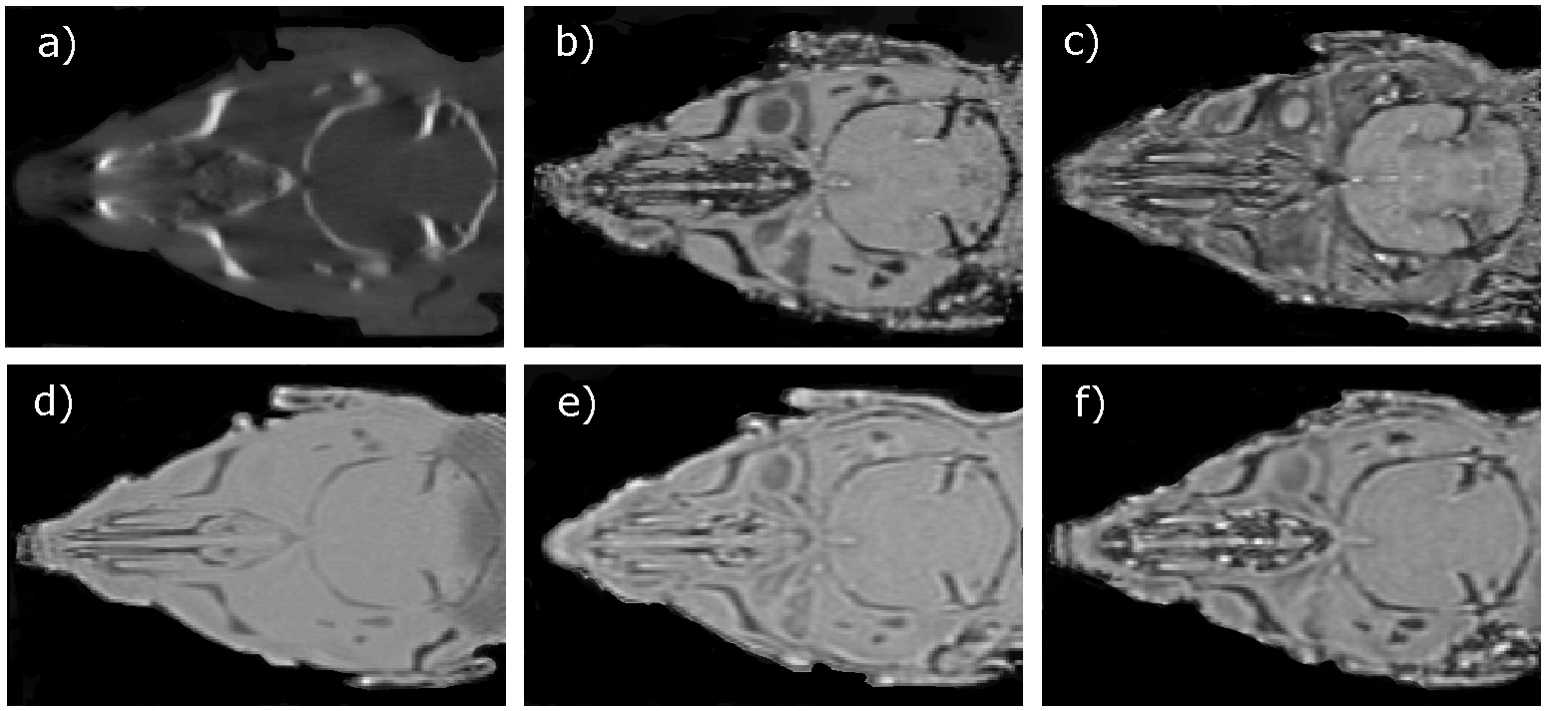
Illustration of a CB-CT and five MR images of the same animal. a) Coronal CB-CT image and (b)-(f) biased field corrected and masked coronal MR images using a T1-weighted (b), T2-weighted (c), ZTE (d), UTE1 (e) and UTE2 (f) sequence.

Following MR image acquisition, the animals were moved to a Small Animal Radiation Research Platform (SARRP) (XStrahl, Surrey, UK) using the in-house made multi-modality bed to maintain a fixed rat head position. A CB-CT was acquired with the following parameters: 70kVp, 1mA, 720 projections, 360° rotation and 1mm aluminum filtration resulting in 127s total acquisition time. The acquired CB-CT projection data were reconstructed using a modified Feldkamp reconstruction algorithm into a 411x251x411 matrix with 275μm voxel size. In Fig 2, coronal slices through the head of one of the rats are shown using the six different image acquisitions.

### Image Co-registration

All CB-CT images were first co-registered using 3D Slicer version 3.6.3 (http://www.slicer.org) by rigid body transformations using normalized mutual information. Then, the T1-weighted images of each animal were resampled and manually co-registered, using anatomical landmarks, to the CB-CT by rigid body transformations, which is sufficient because the rat head was fixed using a multi-modality bed. Next, the other MR images were resampled and co-registered to the CB-CT using the same transformation parameters used to co-register the T1-weighted images to the CB-CT. Finally, all images were cropped to obtain the same matrix size for each image.

### Image Segmentation

CB-CTs were manually segmented into three tissue classes (air, soft tissue and bone) in accordance with the standard method on the SARRP for brain tumors. These segmented CB-CT images were used as a reference for further dose calculations using the treatment planning software (TPS) on the SARRP (3D Slicer Version 3.6.3). MR images were automatically segmented in the same three tissue classes using a modified version of fuzzy c-means segmentation (FCM) algorithm [15], written in MATLAB version R2011b (MathWorks, Natick, MA). FCM is a data clustering technique in which a dataset X = {x_1_…x_n_} is grouped into c clusters C = {c_1_…c_n_} by minimizing the following objective function:

$$Q=\sum_{i=1}^{n}\sum_{j=1}^{c}u_{ij}^{m}\|x_{i}-c_{j}{\|}^{2}$$

Here, || || is the norm expressing the similarity between measured data and cluster. u_ij_ the membership probability of data point x_i_ to belong to cluster c_j_, with the constraint that $\sum_{j=1}^{c}u_{ij}=1$. m is the fuzzification coefficient and is commonly set to 2. To obtain an automatic segmented MR (ASMR) image, voxels were assigned to the tissue class having the highest membership probability. All possible combinations of the five MR images were used as input to the FCM algorithm, with the input image number ranging from one to five. The order of the input images was not relevant, therefore, 31 different ASMR images were obtained. Finally, air masks were generated based on the T1-weighted images to eliminate artifacts outside the rat head.

### Similarity Index

Similarity between segmented CB-CT and ASMR images was evaluated using the 3D Dice coefficient [16]:

$$D=\frac{2\left|CTt\cap MRt\right|}{\left|CTt\right|+\left|MRt\right|}$$

where CTt and MRt represents the voxels classified as tissue class t in segmented CB-CT and ASMR images, respectively. Dice coefficients can be between 0 and 1, with 1 indicating identical segmentation for tissue class t. It was calculated for bone and soft tissue independently because both tissue types are essential for RTP. The average of both Dice coefficients was calculated as the final measure for similarity. ASMR images with high similarity index is used for further RTP, together with the UTE1-UTE2 combination that has already been investigated as a method to generate CT information from MR images [9,12].

### Radiation Therapy Treatment Planning

Manually segmented CB-CT, ASMR, T1- and T2-weighted MR images were imported into the TPS of the SARRP. The target for RTP was selected in the primary motor cortex (M1) using the T1- and T2-weighted images. Three different beam arrangements were investigated to compare CB-CT and MR-based dose calculations. Dose plans were calculated to deliver 15Gy to the target using: (1) a single static beam of 3x3 mm, (2) a single coplanar arc (3x3 mm beam size, 120° arc, couch at 0°), and (3) three non-coplanar arcs (3x3 mm beam size, 120° arc, couch at 0°, 45°, and 90°). For each animal, dose distributions were calculated using the TPS of the SARRP and cumulative dose volume histograms (DVHs) of the target were obtained for the three different beam arrangements using CB-CT and MR-based dose calculations. The target volume was defined as a spherical volume-of-interest around the target with a diameter of 2.5mm. D_5_, D_50_ and D_90_ were calculated for the different dose plans, where D_x_ stands for the dose received by x % of the volume.

### Statistical Analysis

Statistical analysis of D_5_, D_50_ and D_90_ of CB-CT and MR-based dose calculation was performed using the Mann-Whitney U test. A probability value p < 0.05 was considered statistically significant.

## Results

**Fig 3:**
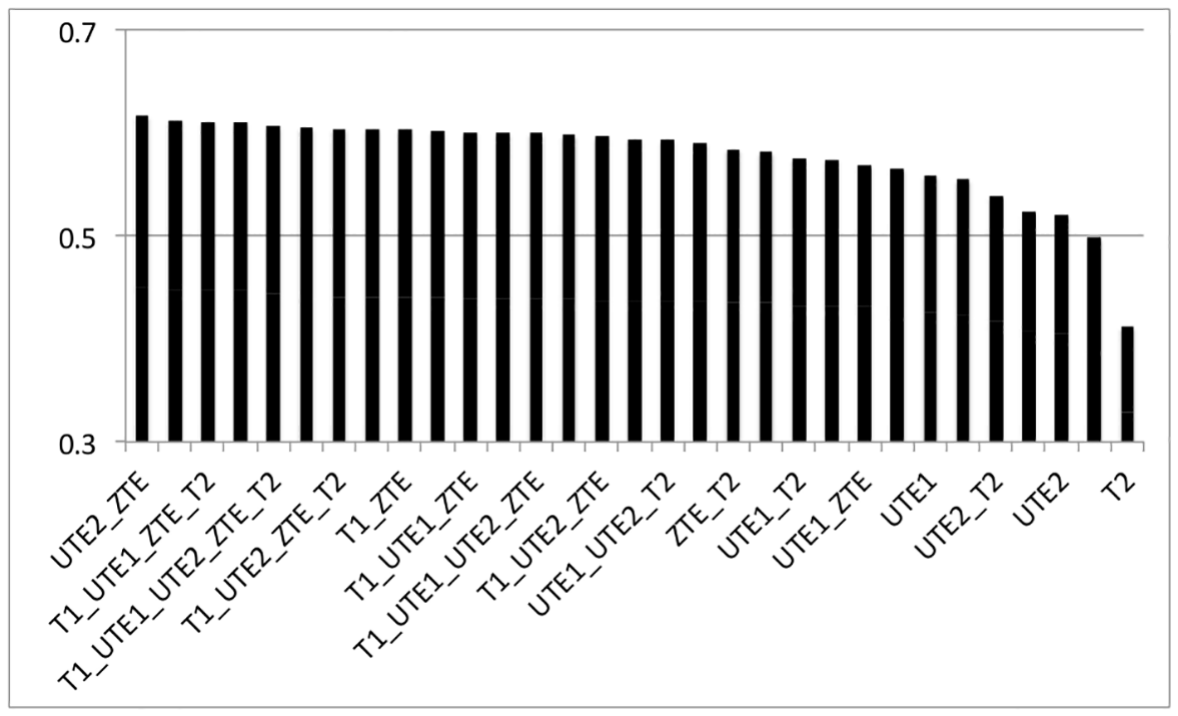
Average Dice coefficient results. Average Dice coefficient for bone and soft tissue segmentation of 31 different combinations of MR-sequences using the CB-CT as a reference.

**Fig 4:**
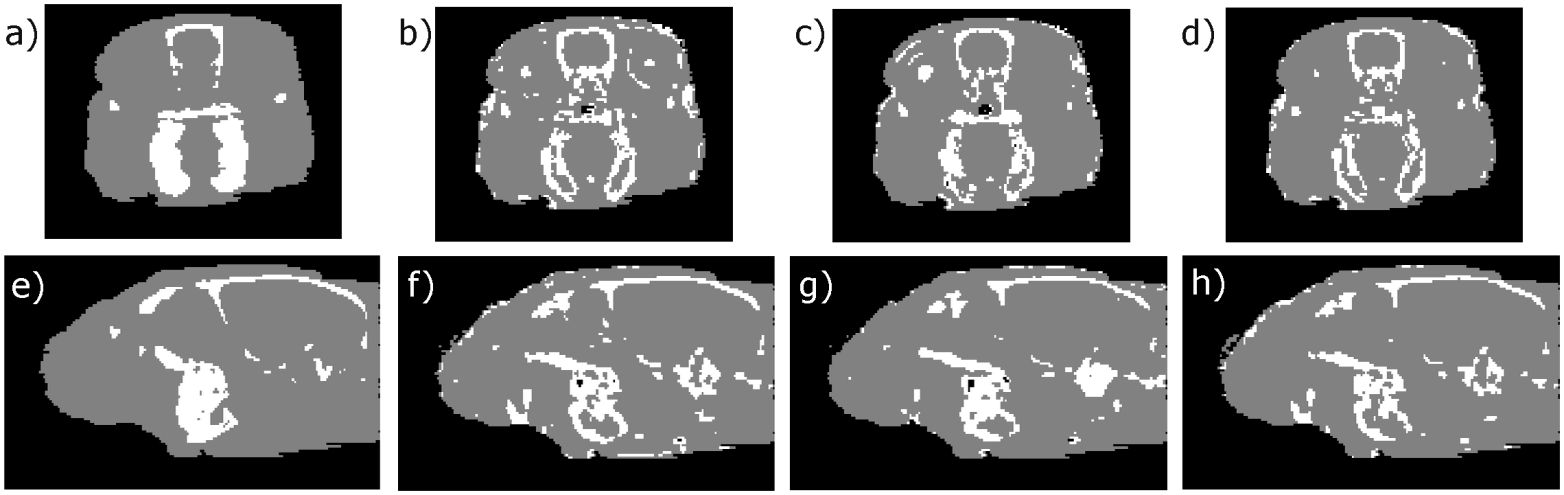
Segmented images into three tissue classes. Manually segmented CB-CT and ASMR images into three tissue classes (air = black, soft tissue = gray and bone = white). Top: Transaxial slice through the rat brain on CB-CT (a), UTE1-UTE2 (b), T1-UTE1-T2(c) and ZTE-UTE2 (d). Bottom: Sagittal slice trough the brain on CBCT (e), UTE1-UTE2 (f), T1-UTE1-T2 (g) and ZTE-UTE2 (h).

Fig 3 shows the similarity between segmented CB-CT and 31 ASMR images obtained by using the 31 possible combinations of the five MR sequences as inputs to the FCM algorithm. The highest Dice coefficient was obtained for the ZTE-UTE2 combination with a value of 0.617±0.013. The highest Dice coefficient for a combination without ZTE was obtained for the T1-UTE1-T2 combination with a value of 0.608±0.019. The average Dice coefficient obtained for the UTE1-UTE2 combination was 0.601±0.013. These three combinations are used for further RTP. Fig 4 shows the result of image segmentation into three tissue classes using CB-CT images and the three MR combinations that will be used for further RTP.

The lowest Dice coefficients were obtained when only one MR image was used as an input to the FCM algorithm and for combinations that did not include a UTE1 and/or ZTE sequence.

**Fig 5:**
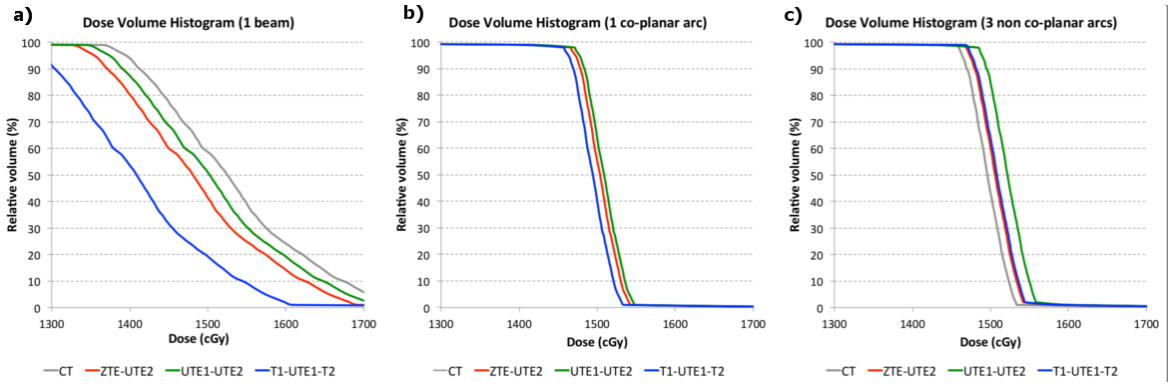
DVHs calculated in the target volume. CB-CT (gray), ZTE-UTE2 (red), UTE1-UTE2 (green) and T1-UTE1-T2 (blue) based dose calculation using (a) 1 beam, (b) 1 co-planar arc and (c) 3 non-coplanar arcs.

Fig 5 displays the DVHs, averaged over the six animals, for CB-CT and MR-based dose calculations in the target volume. Results are shown for the three different beam arrangements.

The D_5_, D_50_ and D_90_ values normalized to the CB-CT are shown in Table 2. In case of the 1-beam configuration, a significant lower dose was delivered to the target when the T1-UTE1-T2 combination was used to generate ASMR images for dose calculations. When 1 arc or 3 arcs were used, a significant higher dose was delivered to the target volume when the UTE1-UTE2 combination was used to generate ASMR images for dose calculations. For the three different beam configurations, no significant difference was observed between dose calculations based on the ZTE-UTE1 combination and CB-CT.

**Table 2:** D_5_, D_50_ and D_90_ values, in the target volume, normalized to the CB-CT reference for the three MR based dose calculations and for three different beam configurations.

	ZTE-UTE1 based dose calculations	T1-UTE1-T2 based dose calculations	UTE1-UTE2 based dose calculations
**1 beam**	0.972±0.064	0.924±0.076*	0.986±0.061
**1 arc**	1.006±0.008	1.000±0.006	1.010±0.005*
**3 arcs**	1.007±0.008	1.008±0.013	1.017±0.012*

Results are shown as mean±sd. Values that are significantly different from the CB-CT reference are marked with an asterisk.

## Discussion

To achieve accurate target volume image guided irradiation, SARRP’s onboard CB-CT guidance is insufficient for the rodent brain because brain tumors will be hardly visible due to the inappropriate soft tissue contrast [6]. The use of contrast CT imaging might offer higher accuracy. However, in the case of brain tumors only late-stage or high-grade tumors might be visualized and there is a significant concern that a high atomic number contrast agent may produce errors in the dose calculation. Consequently, MR-guided radiotherapy is an attractive alternative that allows better target delineation. However, MR images cannot be used for dose planning, as they do not provide the required electron density information.

Therefore, a segmentation method based on the FCM algorithm, with multiple MR sequences as inputs, has been presented and evaluated in the rat brain. The ASMR images presented were sufficiently similar to the manually segmented CB-CT images that are routinely used in small animal image guidance and RTP [1,2]. The Dice coefficient was used to measure the similarity between CB-CT and ASMR images.

This study confirms the importance of acquiring multiple MR images for accurate dose calculations. The lowest Dice coefficients were obtained when only one MR image was used as an input to the FCM algorithm. Higher Dice coefficients were obtained when an MR-sequence that presents higher signal intensities from bone, such as UTE and ZTE, was combined with a conventional MR-sequence. This observation is consistent with recent findings from Hsu et al. [13]. However, the method presented here differs in that it includes the ZTE sequence and it implements an MR-only based workflow for pre-clinical RTP instead of clinical treatment planning.

We observed that MR combinations, including the ZTE sequence, resulted in the highest Dice coefficients. The ZTE sequence, due to its zero echo time, offers higher signal intensities from bone than UTE images. Thus it allows a better differentiation between air and bone.

When dose distributions were calculated using the ASMR images, DVHs indicated small differences with CB-CT based dose calculations. Using only one beam, MR based RTP tends to underestimate the dose to be delivered to the target (range: 1.4%-7.6%). The front part of rat skull (rostrum) is a pretty complex structure that is challenging to segment correctly using MR images. Consequently, when only 1 beam is directed through the front part of skull to reach the brain, the MR based dose calculation might become less accurate. If multiple beams are used that are directed into multiple directions, this possible inaccuracy will be less pronounced. In multiple beam configurations the smallest differences to CB-CT based dose calculations were obtained using the ZTE-UTE2 and T1-UTE1-T2 combinations. The UTE1-UTE2 combination overestimated the delivered dose (range: 1.7%) that is most likely explained because parts of the rat head air cavities and the eyes can be misclassified as bone.

This study has primarily been concerned with the accuracy of MR-based dose calculations for RTP in small animal radiation research; however, total scanning length might become an issue related to animal anesthesia and to provide high throughput. The scan duration of a high-quality ZTE-UTE2 sequence is in the order of 40 minutes. Therefore, total MR scan time reduction should be further investigated to be able to implement it in a daily protocol. CB-CT acquisitions are generally much faster and less expensive than MR. However, one of the most challenging aspects of CB-CT imaging relates to the radiation dose received by the animals. Measurements have shown typical radiation doses in the range of 10–50 cGy for a single CB-CT [17]. The dose at which 50% of mice die within 30 days (LD50/30) is roughly 5 Gy, so a single CB-CT can represent as much as 10% of the LD50/30. This might become very important when delivering the therapeutic dose in multiple fractions spaced over time using CB-CT guided RTP.

### Study Limitations

Our study was limited to the rat brain and an in-house made multi-modality bed was sufficient to simplify the co-registration of CBCT and MR images to rigid body transformations. However, further research is needed before translating these results to other parts of the body. In the rat brain, segmentation into three tissue classes might be sufficient to allow accurate dose calculations. However, more tissue classes will be necessary to provide accurate dose calculations in the thoracic or abdominal region of small animals [18, 19].

Furthermore, the proposed MR-only based workflow still uses CB-CT for animal positioning. In case of an MR-only based workflow a solution should be found to ensure a common coordinate system between MR image space and SARRP irradiation space, which is a non-trivial issue. Possible solutions are the use of digitally reconstructed radiographs extracted from the MR images, using ultra-fast low-dose CB-CT images to perform CB-CT/MR co-registration or the integration of an optical system in combination with a robust animal fixation system.

## Conclusion

We presented an MR-only based workflow for rat brain radiotherapy treatment planning on a small animal radiation research platform that enables both accurate organ delineation and accurate dose calculations using multiple MR images. We have demonstrated that MR-based dose calculations can be improved by including the ZTE sequence.

## References

[1] Verhaegen F, Granton P, Tryggestad E. Small animal radiotherapy research platforms. Phys Med Biol. 2011; 56:R55–R83. 10.1088/0031-9155/56/12/R01 21617291

[2] Vanhoof SJ, Granton PV, Verhaegen F. Development and validation of a treatment planning system for small animal radiotherapy: SmART-Plan. 2013;361–66. 10.1016/j.radonc.2013.10.003 24183860

[3] Aird E, Conway J. CT Simulation for radiotherapy treatment planning. Br J Radiol. 2002;75:937–49. 12515702 10.1259/bjr.75.900.750937

[4] Siewerdsen JH, Moseley DJ, Bakhtiar B, Richard S, Jaffray DA. The influence of antiscatter grids on soft-tissue detectability in cone-beam computed tomography with flat-panel detectors. Med Phys. 2004;31:3506–20. 15651634 10.1118/1.1819789

[5] Fiorentino A, Caivano R, Pedicini P, Fusco V. Clinical target volume definition for glioblastoma radiotherapy planning: magnetic resonance imaging and computed tomography. Clin Transl Oncol. 2013;754–58. 10.1007/s12094-012-0992-y 23359180

[6] Bolcaen J, Descamps B, Deblaere K, Boterberg T, Hallaert G, Van den Broecke C et al MRI-guided 3D conformal arc micro-irradiator of a F98 glioblastoma rat model using the Small Animal Radiation Research Platform (SARRP). J Neuroonc. 2014; 25069566 10.1007/s11060-014-1552-9

[7] Figueroa SD, Winkelmann CT, Miller HW, Volkert WA, Hoffman TJ. TLD assessment of mouse dosimetry during microCT imaging TLD assessment of mouse dosimetry during microCT imaging. Med Phys. 2008;3866–74. 18841837 10.1118/1.2959847 PMC2809703

[8] Shukla D, Huilgol NG, Trivedi N, Mekala C. T2 weighted MRI in assessment of volume changes during radiotherapy of high grade gliomas. J Cancer Res Ther. 2005;235–38. 17998661 10.4103/0973-1482.19601

[9] Keereman V, Fierens Y, Broux T, De Deene Y, Lonneux M, Vandenberghe S. MRI-Based Attenuation Correction for PET/MRI Using Ultrashort Echo Time Sequences. J Nucl Med. 2010; 51:812–8. 10.2967/jnumed.109.065425 20439508

[10] Johansson A, Garpebring A, Karlsson M, Asklund T, Nyholm T. Improved quality of computed tomography substitute derived from magnetic resonance (MR) data by incorporation of spatial information-potential application for MR-only radiotherapy and attenuation correction in positron emission tomography. Acta Oncol. 2013;1369–73. 10.3109/0284186X.2013.819119 23984810

[11] Eilertsen K, Vestad LN, Geier O, Skretting A. A simulation of MRI based dose calculations on the basis of radiotherapy planning CT images. Acta Oncol. 2008; 1294–302. 10.1080/02841860802256426 18663645

[12] Johansson A, Karlsson M, Nyholm T. CT substitute for MRI sequences with ultrashort echo time. Med.Phys.2011;5:2708–14 10.1118/1.3578928 21776807

[13] Hsu SH, Cao Y, Huang K, Feng M, Balter JM. Investigation of a method for generating synthetic CT models from MRI scans of the head and neck for radiation therapy. Med Phys. 2013; 58:8419–8435. 10.1088/0031-9155/58/23/8419 PMC3886820 24217183

[14] Li C, Xu C, Anderson AW, Gore JC. MRI tissue classification and bias field estimation based on coherent local intensity clustering: a unified energy minimization framework. Inf Process Med Imaging. 2009;21:288–99. 19694271 10.1007/978-3-642-02498-6_24

[15] Bezdek JC. Pattern Recognition with Fuzzy Objective Function Algorithms. Kluwer Academic Publishers Norwell, MA, USA 1981. ISBN:0306406713.

[16] Sørensen T. A method of establishing groups of equal amplitude in plant sociology based on similarity of species and its application to analyses of the vegetation on Danish commons. Kongelige Danske Videnskabernes Selskab. 1948;1–34.

[17] Cherry SR. In vivo molecular and genomic imaging: new challenges for imaging physics. Phys Med Biol 2004:49:R13–R48. 15012005 10.1088/0031-9155/49/3/r01

[18] Bazalova M, Graves EE. The importance of tissue segmentation for dose calculations for kilovoltage radiation therapy. Med Phys 2011;38:3039–3049. 21815377 10.1118/1.3589138 PMC3125081

[19] Verhaegen F, van Hoof S, Granton PV, Trani D. A review of treatment planning for precision image-guided photon beam pre-clinical animal radiation studies. Z Med Phys 2014;24:323–334. 10.1016/j.zemedi.2014.02.004 24629309

